# Birth weight and characteristics of endothelial and smooth muscle cell cultures from human umbilical cord vessels

**DOI:** 10.1186/1479-5876-7-30

**Published:** 2009-04-24

**Authors:** José Javier Martín de Llano, Graciela Fuertes, Isabel Torró, Consuelo García Vicent, José Luis Fayos, Empar Lurbe

**Affiliations:** 1 Laboratory of the Pediatric Cardiovascular Risk Unit, Pediatric Department, Consorcio Hospital General Universitario de Valencia, and CIBER Fisiopatología de la Obesidad y Nutrición (Instituto de Salud Carlos III), Spain; 2 Clinic of the Pediatric Cardiovascular Risk Unit, Pediatric Department, Consorcio Hospital General Universitario de Valencia, and CIBER Fisiopatología de la Obesidad y Nutrición (Instituto de Salud Carlos III), Spain

## Abstract

**Background:**

Low birth weight has been related to an increased risk for developing high blood pressure in adult life. The molecular and cellular analysis of umbilical cord artery and vein may provide information about the early vascular characteristics of an individual. We have assessed several phenotype characteristics of the four vascular cell types derived from human umbilical cords of newborns with different birth weight. Further follow-up studies could show the association of those vascular properties with infancy and adulthood blood pressure.

**Methods:**

Endothelial and smooth muscle cell cultures were obtained from umbilical cords from two groups of newborns of birth weight less than 2.8 kg or higher than 3.5 kg. The expression of specific endothelial cell markers (von Willebrand factor, CD31, and the binding and internalization of acetylated low-density lipoprotein) and the smooth muscle cell specific α-actin have been evaluated. Cell culture viability, proliferation kinetic, growth fraction (expression of Ki67) and percentage of senescent cells (detection of β-galactosidase activity at pH 6.0) have been determined. Endothelial cell projection area was determined by morphometric analysis of cell cultures after CD31 immunodetection.

**Results:**

The highest variation was found in cell density at the confluence of endothelial cell cultures derived from umbilical cord arteries (66,789 ± 5,093 cells/cm^2 ^vs. 45,630 ± 11,927 cells/cm^2^, p < 0.05). Morphometric analysis indicated that the projection area of the artery endothelial cells (1,161 ± 198 and 1,544 ± 472 μm^2^, p < 0.05), but not those derived from the vein from individuals with a birth weight lower than 2.8 kg was lower than that of cells from individuals with a birth weight higher than 3.5 kg.

**Conclusion:**

The analysis of umbilical cord artery endothelial cells, which demonstrated differences in cell size related to birth weight, can provide hints about the cellular and molecular links between lower birth weight and increased adult high blood pressure risk.

## Background

There is increasing interest in knowledge about the impact of intrauterine development during adult life [1]. Low growth rate in fetal life is associated with increased death rates from coronary heart disease and stroke [2-5]. Hypertension is a risk factor for ischaemic heart disease and stroke [5] and hypertension has been suggested as one link between intrauterine environment and risk of cardiovascular disease [6]. In previous studies an inverse relationship between birth weight and blood pressure (BP) levels has been demonstrated in babies who are "small for date" rather than in those born prematurely [7-9]. Furthermore, low birth weight has also been associated with alterations of vascular function in children and adolescents [10].

The impact of intrauterine life in the newborn period has been demonstrated [11]. Low birth weight individuals showed a lower systolic BP and a steeper increase of the systolic BP during the first month after birth than did individuals that showed a higher weight at birth. The direct association at birth and the inverse association at one month of life point out that the association between birth weight and BP reverses direction during this time period. The steepest BP increase was observed in children with intrauterine growth retardation. Whether or not changes in BP in low birth weight subjects may result from vascular imprinting with early changes in cells from the vascular wall is an intriguing question. We hypothesize that it will be possible to find vascular cell phenotypes that could be associated with birth weight. These findings may provide hints of the link to adult BP, through molecular changes, as epigenetic modifications that can influence vascular development. Therefore, umbilical cord (UC) vessels can be useful in order to detect differential phenotypes since vascular wall cells experience the effect of hormonal and hemodynamic changes, which occur during fetal life period.

The study of endothelial and smooth muscle cells from UC vessels can help to look for the alterations involved in the functional vascular changes associated with lower birth weight. Of the UC vessels, the vein is a classic source of both endothelial and smooth muscle cells (EC and SMC, respectively), mostly because it is a large vessel that can be easily handled [12]. Umbilical arterial vessels, however, have been used as a source of EC and SMC less frequently since their small diameter makes handling difficult [13-15] even if they are a vascular bed prone to reflect early changes in fetal life due to its directly receiving the impact of the fetal milieu. The UC is an exceptional source of vascular cells, which can offer valuable information about the cellular characteristics of the blood vessels of the individual and their relationship with properties of the vascular system, such as blood pressure. To our knowledge, there are not previous studies about the link between birth weight and the properties of the cells from the UC vessels. Our aim has been obtaining the four vascular cell types from each individual UC to determine their cellular and molecular properties, as both ECs and SMCs are important in maintaining the vascular tone.

We have recently developed a suitable procedure to routinely obtain EC and SMC cultures from both the vein as well as the arteries of an individual's UC [16]. The objective of the present study was to assess simultaneously several phenotype characteristics of the four cellular types derived from human UC of newborns with birth weights < 2.8 kg or > 3.5 kg, to gain information about the cellular and molecular links between lower birth weight and increased adult high blood pressure risk.

## Methods

Affinity purified IgG fraction of an anti-human Ki67 antiserum developed in rabbit was from Abcam (Cambridge, UK). Fluorescein isothiocyanate (FITC)-conjugated F(ab')_2 _fragment of anti-rabbit IgG developed in goat, ribonuclease A and ethidium homodimer were from Sigma-Aldrich Inc. (St. Louis, Missouri, USA). 5-bromo-4-chloro-3-indolyl-beta-D-galactopyranoside (X-Gal) was from Eppendorf AG (Hamburg, Germany). The source of the other reagents and materials has been previously described [16].

### UC samples

UC samples were obtained after uncomplicated pregnancies, at term (gestational age ≥ 37 weeks), ascertained according to the method of Ballard et al. [17] and normal delivery or Caesarian section in the absence of perinatal illness, at the Hospital General Universitario de Valencia, Spain. All the mothers were healthy and had no cardiovascular risk factors, except for those who were active smokers. Anthropometric measurements were done as previously described [11]. Two groups of newborns were considered according to birth weight lower than the twenty-fifth percentile (group 1) or higher than the seventy-fifth percentile (group 2) (ie, lower than 2.8 and higher than 3.5 kg, respectively). Parents gave their consent for the study after they were informed of the objectives of the research project and the samples that would be used. The research was carried out according to the principles of the Declaration of Helsinki, and the study was approved by the hospital's review board.

### UC arteries and vein endothelial and smooth muscle cell isolation

A segment of the UC was clamped at both ends, severed and kept at 4°C for a maximum of 24 h in sterile Hank's Balance Salt Solution containing 100 unit/mL penicillin and 100 μg/mL streptomycin. ECs and SMCs from UC arteries and vein were obtained and cultured as described [16]. Human umbilical arteries or vein ECs (HUAECs and HUVECs, respectively) were harvested after enzymatic treatment by incubation of the corresponding vessel lumen with a collagenase-dispase mixture and cultured on flasks coated with fibronectin using an optimized EC culture media. The human umbilical arteries or vein SMCs (HUASMCs and HUVSMCs, respectively) were obtained from explants of the corresponding vessels after removing the ECs as described above and cultured on dishes or flasks coated with collagen using an optimized SMC culture media. Subclonfluent primary ECs or SMCs cultures covering a 75-cm^2 ^growing area were harvested and 3 aliquots cryopreserved. These aliquots were considered to correspond to cells at passage 0.

### Cellular characterization

Cryopreserved ECs or SMCs were thawed and cultured on flasks, dishes, plates or glass coverslips coated with fibronectin or collagen, respectively. Culture media was changed every 48 hours. Subconfluent cultures were split 1:3. When required, cell number was calculated by counting harvested cells using a hemocytometer chamber.

#### Cell viability and cellular proliferation

Passage 2–4 cells were seeded at 10,000 cell/cm^2 ^on 12 mm diameter glass coverslips placed in 24-well plates. Viability was assessed after 3 days by the Trypan blue exclusion test, counting Trypan blue-stained and total number of cells as previously described [16].

Cells were seeded in 96-well plates at 10,000 cell/cm^2 ^in 150 μL cell culture media/well, and incubated as above. A plate was removed from the incubator every 24 hours. The cell culture media from this plate was removed by blotting on a stack of paper sheets. An excess of Dulbecco's phosphate-buffered saline (DPBS) warmed to 37°C, was added onto the wells and quickly removed by blotting the plate again. Blotted plates were kept at -80°C until the assay. The complete set of plates from a proliferation experiment were allowed to warm up to room temperature and 150 μL of DPBS containing 0.7 units of DNase-free ribonuclease A was added to each well. After 60 min incubation at 37°C, 50 μL of 8 μM ethidium homodimer and 0.4% saponin solution in DPBS was added. The plates were incubated in the dark at room temperature for 45 min and the light emitted was measured in a Victor3 1420 Multilabel Counter (excitation and emission filters of 530 and 616 nm, respectively). A standard cell suspension of every cell type was prepared in DPBS and kept at -80°C until use.

The growth fraction of exponentially growing or confluent HUAEC cultures was estimated determining the percentage of cells expressing Ki67 (see below) from the total number of cells.

### Cellular markers

The expression of von Willebrand (vW) factor, CD31 (platelet endothelial cell adhesion molecule-1, PECAM-1), Ki67 and the SMC specific α-actin was determined in cells grown on circular coverslips by indirect immunofluorescence as described [16]. Cells were fixed and incubated with the corresponding primary antibody and subsequently with a matching secondary antibody conjugated to tetramethylrhodamine isothiocyanate (TRITC), for vW factor detection, or FITC, for Ki67, CD31 and α-actin immunodetection. The microscope slide was placed in a Leica DM 6000 B fluorescence microscope to which a Leica DFC 480 digital camera system was connected. TRITC or FITC positive and total number of cells, as assessed by cells visualized by Differential Interference Contrast (DIC) or 4',6-diamidino-2-phenylindole dihydrochloride (DAPI)-stained nucleus were counted from matching images. To estimate the number of ECs that could be present in a SMC culture, the total number of vW factor positive cells from 2 coverslips was counted. To estimate the number of SMCs that could contaminate an EC culture, the total number of α-actin positive cells from 2 coverslips was counted.

CD31 preparations were used to measure EC projection area of confluent cultures. Merged images of several randomly selected areas were obtained using a 40× objective as described above and analyzed using the Leica IM500 image manager software. The average percentage distribution of the ECs projection area was calculated from the area data of 50 cells from each EC culture included in the corresponding study. Aberrant multinucleated cells were excluded from the distribution analysis. The binding and internalization of Ac-LDL was determined by incubating cells grown on circular coverslips with culture media containing 1,1'-dioctadecyl-3,3,3',3'-tetramethylindocarbocyanine perchlorate (DiI)-labeled Ac-LDL as described [16].

#### Cellular senescence

Cells were seeded as above, and the percentage of senescent cells was determined as follows. Cell culture media was removed from the well and 1 mL of DPBS at room temperature was added. After 1 min DPBS was removed and cells were fixed for 3 min with 1 mL of 3% paraformaldehyde in DPBS at room temperature. The solution was removed and cells were washed twice with 2 mL of DPBS. The senescence assay was then carried out as described [18], incubating the fixed cells for 16 h at 37°C in a citric acid-sodium phosphate pH 6.0 solution containing the β-galactosidase substrate X-Gal. The coverslip was placed on a microscope slide and the cell monolayer covered with a drop of FA mounting fluid pH 7.2 containing 1.25 μg/mL DAPI. Several images of randomly selected areas were recorded using a 10× lens under both bright field, as well as fluorescence conditions. Senescence (blue-stained cells observed under bright field conditions) and total number of cells (DAPI-stained nucleus observed under fluorescence conditions) were counted from matching images.

### Statistical analysis

Experimental values are expressed as mean ± SD. Differences between groups were evaluated with Student's t-test, Mann-Whitney test, or χ^2 ^test, as appropriate. A significant difference was considered present if p < 0.05. For the HUAEC projection area determination, sample size was estimated considering that the assay could detect (significance level 0.05, 80% power) a difference between means of the 2 groups corresponding to 25% of the mean projection area calculated in a pilot study (1,300 ± 250 μm^2^). Statistical analyses were performed using SPSS 13.0 (SPSS Inc, Chicago, Illinois, USA) and GraphPad Statmate 2.0 (GraphPad Software, La Jolla, California, USA) softwares.

## Results

### Characteristics of the study population

Table 1 shows the general characteristics of the study groups. There were no significant differences in the type of delivery, sex distribution, gestational age and maternal smoking habit between the two birth weight groups. The <2.8 kg birth weight group (group-1) had systolic and diastolic BP values significantly lower than the >3.5 kg birth weight group (group-2), as expected [11].

**Table 1:** General characteristics of the study sample grouped by birth weight

	Birth weight <2.8 kg	Birth weight >3.5 kg
Number of babies	11	11
Delivery (normal/Caesarian section)	6/5	9/2
Sex (male/female)	4/7	8/3
Gestational age (weeks)	38.3 ± 1.6	39.3 ± 1.0
Mother smoker (no/yes)	7/4	9/2
Weight (g)	2612 ± 188	3999 ± 379
Systolic BP (mmHg)*	65.9 ± 10.1	76.7 ± 5.7
Diastolic BP (mmHg)†	42.0 ± 7.5	47.8 ± 4.4

*Statistically significant difference between groups (p < 0.01)

†Statistically significant difference between groups (p < 0.05)

### Characterization of the cell types and growth kinetics of cultured cells

Healthy growing EC and SMC cultures were obtained from UC of group-1 and group-2 individuals. No contamination of SMCs in EC cultures was observed, and a low average level (<0.009%) of EC contamination of SMC cultures was observed, assessed considering the binding and internalization of DiI-labeled Ac-LDL. Average time to reach passage 0 cell density and percentage of viable cells values were similar to those previously described [16]. Replicative senescence level was slightly higher for HUAEC than for HUVEC cultures (4.5 ± 2.2 and 1.2 ± 0.4%, respectively; p = 0.005).

**Figure 1:**
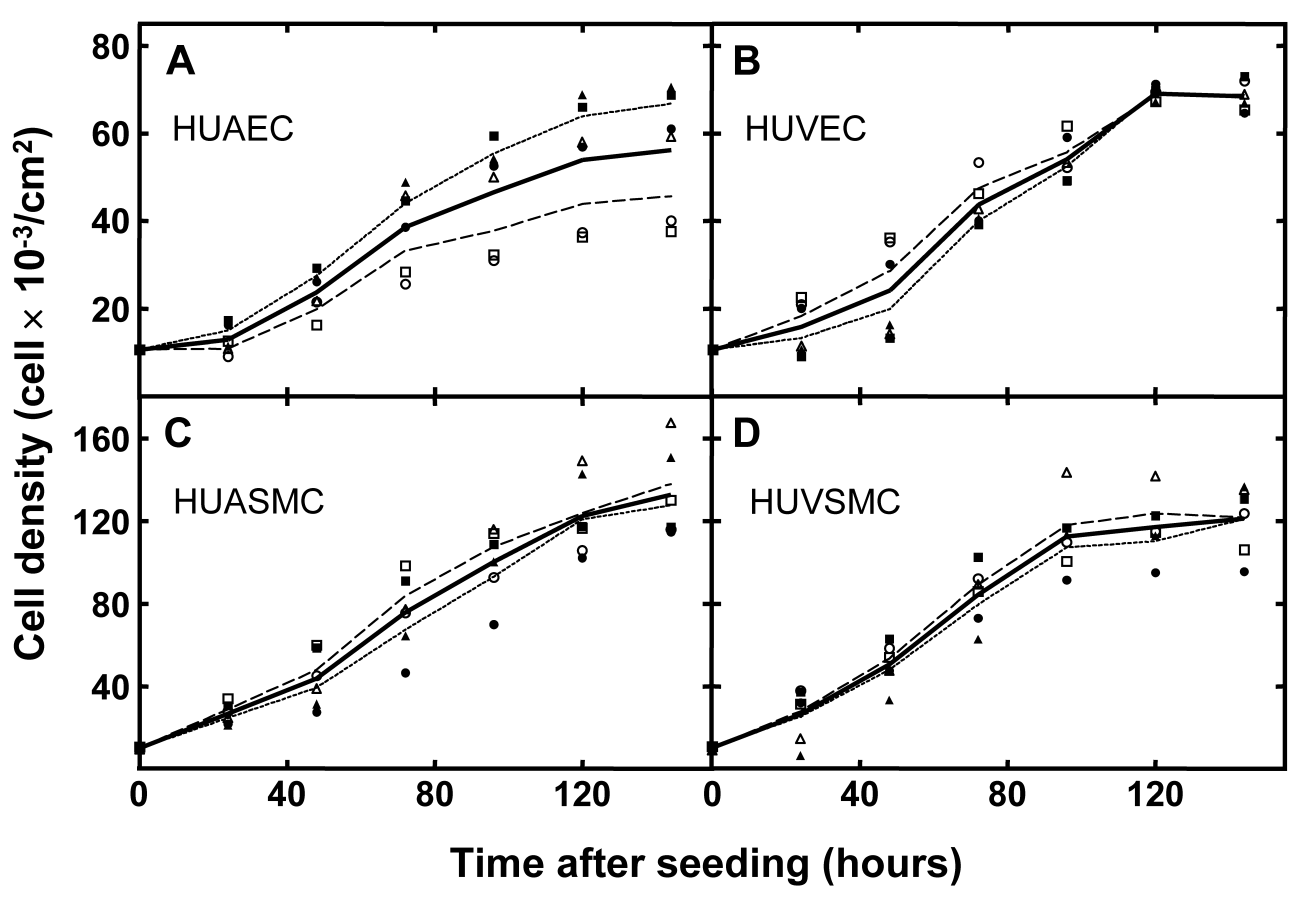
**Cell proliferation kinetics of vascular cell types obtained from human umbilical cords (UCs)**. Human umbilical artery and vein endothelial (HUAECs and HUVECs, panels A and B, respectively) and smooth muscle cells (HUASMCs and HUVSMCs, panels C and D, respectively) obtained from 6 UCs of newborns (birth weight <2.8 kg, n = 3 solid symbols or >3.5 kg, n = 3 hollow symbols) were seeded and cultured as described in Methods. Each experimental point corresponds to the mean of three replicates. In each panel, the lines shown connect the calculated average values from each time point analyzed corresponding to all the individuals (solid line) or to those individuals grouped according to their lower (<2.8 kg, dotted line) or higher (>3.5 kg, dashed line) birth weight in order to facilitate a comparison.

Several growth parameters of the different cell cultures were analyzed. Figure 1 shows the cell proliferation kinetics of the 4 cell types obtained from 6 individuals. Cell culture growth follows the expected behavior. After a lag phase, that is more evident in the HUAEC and HUVEC cultures (Figure 1, panels A and B, respectively solid line) than in HUASMC and HUVSMC cultures (Figure 1, panels C and D, respectively solid line), a logarithmic phase of cell growth follows, leading eventually to a stationary or confluent phase. From the logarithmic growth phase, the average cell population doubling time for every cell type was calculated. According to this data, the average doubling time for HUAEC, HUVEC, HUASMC and HUVSMC were similar (46.1, 47.0, 47.7 and 42.3 h, respectively) and the differences among all of them were not statistically significant. Furthermore, the average number of cells in the confluent phase was estimated; ie, 144 hours after seeding. HUAECs reach a lower cell density at confluence than do HUVECs (56,210 ± 14,198 and 68,461 ± 3,463 cells/cm^2^, respectively), although the difference is not statistically significant (p = 0.067). Both HUASMCs and HUVSMCs reach approximately the same cell density (132,670 ± 21,856 and 121,032 ± 16,821 cells/cm^2^, respectively; p = 0.326), about twice the number of cells at confluence determined for ECs. A higher dispersion of cell density among the HUAEC cultures was observed (Figure 1A).

### Birth weight and growth characteristics of cultured cells

No differences in terms of average time to reach passage 0 cell density, percentage of viable cells and senescence level were found for each cell culture type derived from group-1 or group-2 individuals.

To investigate if there were differences in cell density between the 2 birth weight-groups, data were analyzed according to lower (<2.8 kg, n = 3, Figure 1, solid symbols) or higher (>3.5 kg, n = 3, Figure 1, hollow symbols) birth weight. Dotted and dashed lines connecting the average values calculated for the 2 groups (Figure 1, panels A, B, C and D) are shown to help visualize the different behaviors. There were no significant differences in the doubling time for any of the 4 cell type cultures between group-1 and group-2 individuals. However, when the average density of cells at confluence was compared, a significant difference (p = 0.048) was observed for the HUAECs obtained from group-1 (66,789 ± 5,093 cells/cm^2^) and group-2 (45,630 ± 11,927 cells/cm^2^) individuals.

**Figure 2:**
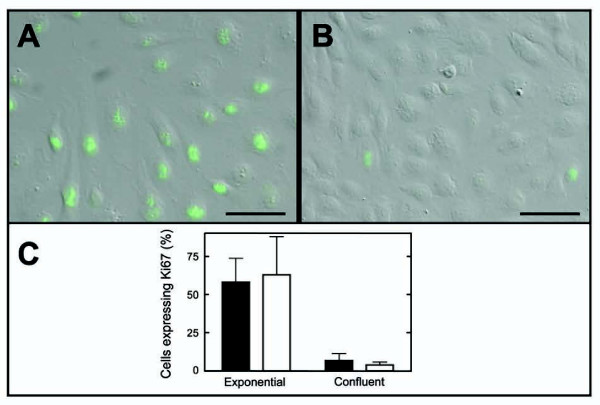
**Proliferation fraction of exponentially growing and confluent human umbilical artery endothelial cell cultures**. Ki67 was detected by indirect immunofluorescence and total number of cells was visualized under differential interference contrast (DIC). Representative merged micrographs of immunofluorescence and DIC images of exponentially growing (A) and confluent (B) cultures are shown. The proliferation fraction of exponentially growing or confluent HUAEC cultures from <2.8 kg (n = 6, black bars) or >3.5 kg (n = 6, white bars) birth weight individuals is shown (C). Differences between the two birth weight groups were not statistically significant. Bar in A and B, 50 μm.

To further characterize the proliferation properties of HUAEC cultures, growth and replicative senescence fractions of exponential growth or confluent cell cultures were determined. No significant difference (p = 0.698) was found between the growth fraction of exponentially growing HUAECs (Figure 2A) from group-1 and group-2 individuals (58.0 ± 15.7 and 62.8 ± 24.9%, respectively, Figure 2C). As expected, the growth fraction dropped when cells reached confluence (Figure 2B). No difference (p = 0.218) was found between group-1 and group-2 individuals (6.8 ± 4.7 and 4.1 ± 1.8%, respectively, Figure 2C). The percentage of senescent cells in exponentially growing HUAEC cultures from group-1 and group-2 were not statistically different (2.7 ± 2.6 and 1.3 ± 0.7%, respectively; p = 0.236). The fraction of senescent cells increased in confluent HUAEC cultures, but no significant differences were observed between cells from group-1 and group-2 individuals (4.2 ± 3.0 and 4.9 ± 4.6%, respectively; p = 0.761).

### Birth weight and HUAEC projection area

**Figure 3:**
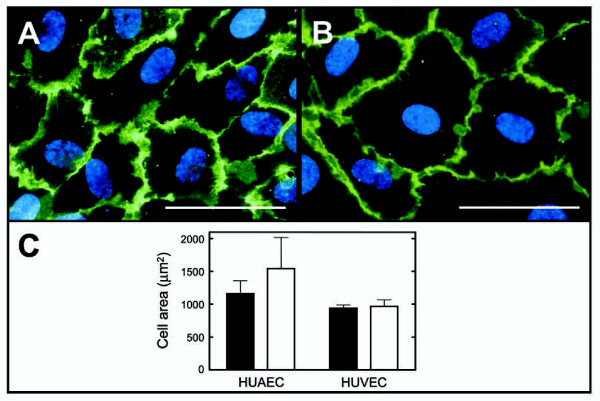
**Projection area of human umbilical artery and vein endothelial cells grown to confluence**. Passage 2–4 HUAECs and HUVECs were grown to confluence and fixed. CD31 was localized by indirect immunofluorescence, and DNA was labeled with 4',6-diamidino-2-phenylindole dihydrochloride. The projection area of 50 cells was calculated (see Methods). A and B are representative merged micrographs of HUAECs from a <2.8 kg or >3.5 kg birth weight individual, respectively, showing the presence of CD31 in the cell perimeter, as well as the cell nucleus. C, projection area of HUAECs and HUVECs from individuals of <2.8 kg (n = 11 and n = 6, respectively, black bars) or >3.5 kg (n = 11 and n = 6, respectively, white bars) birth weight. Difference was statistically significant (p < 0.05) for the area of HUAECs from both groups. Bar in A and B, 50 μm.

To verify if the dissimilar average cell density at confluence of HUAEC cultures was related to cell size, 22 HUAEC cultures were allowed to reach confluence and cell perimeter was visualized through immunodetection of CD31 (Figures 3A and 3B). Twelve HUVEC cultures were also analyzed for comparison purposes. From the morphometric analysis, the average cellular projection area for HUAECs derived from individuals of birth weight <2.8 kg (Figure 3A) or >3.5 kg (Figure 3B) were statistically different from each other, 1,161 ± 198 and 1,544 ± 472 μm^2 ^(Figure 3C), respectively (p = 0.022). No statistically significant differences were found for the HUAEC projection area when samples were grouped according gender (males, n = 12 1,360 ± 382 μm^2^, females, n = 10 1,343 ± 450 μm^2^, p = 0.923) and for the average cellular projection area of HUVECS from group-1 and group-2 (941 ± 51 and 967 ± 100 μm^2^, respectively; p = 0.583).

**Figure 4:**
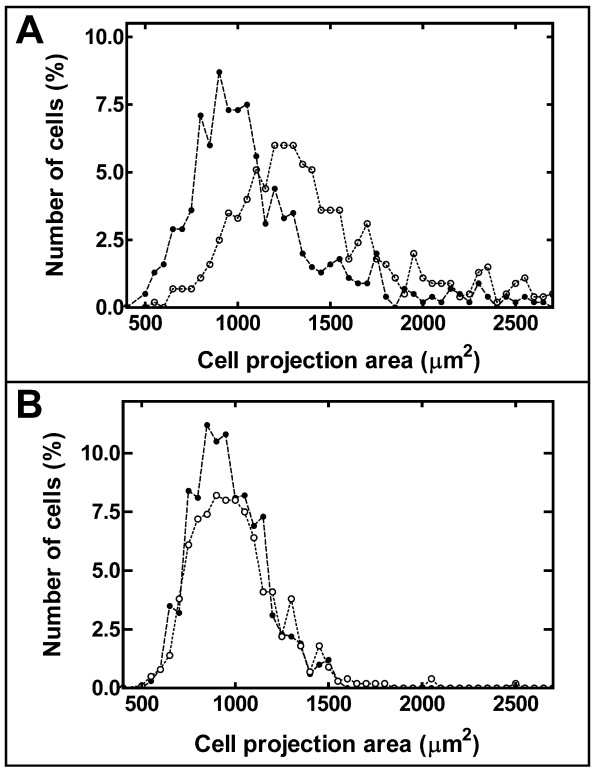
**Average percentage distribution of endothelial cell projection area**. The average percentage distribution of the projection area of human umbilical artery (panel A) and vein (panel B) endothelial cell cultures (see legend from Figure 3C) was calculated as described in Methods. Solid and hollow symbols trace data from individuals with birth weights of <2.8 kg and >3.5 kg, respectively.

To assess if the differences observed were secondary to some methodological bias, the percentage distribution of the ECs projection area was calculated. HUAECs (Figure 4A, average of cells from 11 individuals from each group) and HUVECs (Figure 4B, average of cells from 6 individuals from each group) from both birth weight groups showed a bell-shaped distribution shifted to the higher surface values. As shown in Figure 4A, the distribution curves of HUAECs obtained from the 2 groups of individuals are similar in shape. The differences described above for the mean value and SD of the HUAECs projection area arise because the size of the cells from group-1 individuals (Figure 4A solid symbols) is shifted to lower values than that from cells of group-2 individuals (Figure 4A, hollow symbols), and because the curve is sharper. As shown in Figure 4B, the average distribution curves of HUVECs from group-1 and group-2 (Figure 4B, solid and hollow symbols, respectively) individuals are similar. The differences observed were not dependent on the presence of a large percentage of multinucleated cells, aberrant cells described in EC cultures frequently associated to a giant size, since they were similar not only in HUAEC cultures from group-1 and group-2 individuals (3.2 and 3.8%, respectively), but also in HUVEC cultures (2.8 and 2.1%, respectively).

## Discussion

Simultaneous growth of endothelial and smooth muscle cells from the UC arteries and veins of children born at term showed that artery endothelial cell cultures coming from the lower birth weight group exhibited a different cell density and size at confluence when compared to that from children of higher birth weight. Analyses of the proliferation kinetics show that average cell density at confluence of HUAECs obtained from subjects with low birth weight is about 1.5 higher than that from those of the normal birth weight group. The differences observed in endothelial arterial cells were not present in ECs from vein nor were they in SMCs from arteries or veins.

The differences observed were not artefactual; ie, they did not arise as a consequence of methodological bias in cell separation and culture or of a small number of samples analyzed. The phenotypic identity of a total of 24 EC and SMC cultures analyzed at passage 2–4 has been confirmed using specific molecular markers and no contamination was found in EC cultures by SMCs. The relationship between HUAECs projection area at confluence and birth weight was observed analyzing cells from 22 individuals, a number of samples which minimized the odds of obtaining that result solely by chance.

These findings need to be considered in the scope of the fetal programming hypothesis. After the initial observation of the effect of intrauterine life on the development of hypertension later in life, an important question arises. What are the mechanisms involved? [19] Although many theories have been proposed, hormonal imprinting [20] and structural changes of blood vessels and/or kidney [21] have received the most attention. The hormonal imprinting hypothesis has been supported by the demonstration of low activity levels of 11-beta-hydroxyesteroid dehydrogenase along with high levels of fetal cortisol in rats. The consequent increment of fetal exposure to maternal cortisol can produce imprinting patterns of response in vascular structures and cerebral tissue that persist throughout life, with or without structural changes in the vascular tree.

The presence of early alterations in vascular function has been described in children and adolescents with low birth weight. They are manifested not only as high systolic BP, both office and ambulatory [22], but also as increments in BP variability [23], pulse pressure [24] and early reflecting waves [10]. These intermediate phenotypes are the expression of functional or structural abnormalities that have been established during fetal life. If this imprinting exists, it can be present at birth even though the greatest impact comes later in life.

A recent paper by our group supports this concept [11]. After birth, a rapid rise in BP during the first weeks of life has been observed in children with low birth weight. The steep BP increment during the first month of life, and the persistence of relatively high BP at the end of the first year, indicate that low birth weight children are prone to develop a phenotype that may lead to a progressive increment of BP over time. Consequently, we hypothesized that biological differences can be observed in UC vessels cells and we found phenotypic differences only in HUAECs.

The results indicate that HUAECs derived from UCs of individuals of low birth weight have a lower cell projection area than those from UCs of individuals of higher birth weight. Endothelial cells exhibit an innate heterogeneity, ie, in phenotype, antigen expression, cell size and growth [25,26]. Cell size and the expression of some connexins, components of gap junctions, decrease in ECs of rat caudal arteries as hypertension develops in spontaneously hypertensive rats [27], although a cell size change was not observed in ECs from the aorta [28]. Considering the different approaches of the studies (human vs rat model, endothelial cell culture vs in situ studies), further studies are necessary to verify if changes in the HUAECs size correlate with changes in connexins. A change in cell size and contact area can modify the intercellular density and composition of such connecting channels as gap junctions, altering the diffusion of molecules across the cells [29]. Whether or not the changes in cellular function can modify the vascular response is an intriguing hypothesis.

Altered endothelial cell function is a key factor associated with vascular disorders and is critical in fetal growth and development. Pregnancies affected by diseases such as gestational diabetes are associated with human umbilical vein endothelial dysfunction. Functional abnormalities of calcium handling and nitric oxide production have been described in HUVECs from preeclampsia deliveries [30]. These were maintained during culture *in vitro *and indicate that this may reflect long-term "programming" of the fetal cardiovascular system. So if the cell projection area at confluence of our HUAEC cultures does reflect differences that can be found *in vivo*, this would facilitate the search for a link between birth weight and perinatal, and perhaps adult BP. The results described herein suggest that, from the 4 vascular cell types studied HUAECs are a promising candidate in the search for molecular differences that could explain the increased risk that lower birth weight individuals exhibit of developing high BP later in life.

## Conclusion

Birth weight is related to BP at birth and in adulthood. Our study shows that it is also related to some properties of a specific vascular cell type. These facts could imply that early changes in the properties of endothelial cells could be associated to functional changes and contribute to an individual's BP phenotype later in life.

## Competing interests

The authors declare that they have no competing interests.

## Authors' contributions

EL and JJMDL conceived and designed the study and wrote the manuscript. JJMDL and GF obtained the cell cultures and carried out the molecular and cellular analysis. CGV and JLF informed the parents about the objectives of the research project, did the anthropometric measurements at birth and obtained the UC samples. IT and EL carried out the follow-up of the individuals included in the study.
